# Immediate Loading of Implants with Fixed Rehabilitations in Geriatric Edentulous Patients; Biological Complications

**DOI:** 10.3390/jcm12206548

**Published:** 2023-10-16

**Authors:** Eugenio Velasco-Ortega, Laura Carretero-Barrado, Jesús Moreno-Muñoz, Ivan Ortiz-García, Enrique Núñez-Márquez, José Luis Rondón-Romero, José López-López, Álvaro Jiménez-Guerra, Loreto Monsalve-Guil

**Affiliations:** 1 Faculty of Dentistry, University of Seville, 41004 Sevilla, Spain; evelasco@us.es (E.V.-O.); lauracarreterobarrado@gmail.com (L.C.-B.); je5us@hotmail.com (J.M.-M.); ivanortizgarcia1000@hotmail.com (I.O.-G.); enrique_aracena@hotmail.com (E.N.-M.); jolurr001@hotmail.com (J.L.R.-R.); lomonsalve@hotmail.es (L.M.-G.); 2 Faculty of Medicine and Health Sciences (Dentistry) & Dentistry Hospital, University of Barcelona, 08907 L’Hospitalet de Llobregat, Spain

**Keywords:** immediate loading, totally fixed rehabilitation, geriatric patients, immediate implants

## Abstract

Background: This study aimed to report the outcomes of the immediate loading of implants with fixed rehabilitations in edentulous geriatric patients. Methods: Edentulous geriatric patients were diagnosed with an oral examination, radiographic evaluation, and intermaxillary relations and treated with fixed rehabilitation over several implants. After immediate surgery, the implants were immediately loaded with a fully fixed prosthesis. Results: Twenty-four patients (20 females and 4 males) were treated using a total 210 implants. All patients (100%) had a previous history of periodontitis. Eleven patients (45.8%) were smokers. Eleven patients (45.8%) suffered from chronic medical diseases (i.e., diabetes, cardiovascular diseases). The study’s clinical follow-up period extended for three years, during which thirty-three fixed prostheses were installed over the implants in 24 patients. The average marginal bone loss measured was 1.33 ± 0.17 mm. The success rate of the implants and prosthodontics being placed in this study yielded 98.5% and 97%, respectively. One patient (4.2%) showed some kind of technical complications. Eleven patients (45.8%) showed mucositis, and 25 implants (11.9%) in 10 patients (41.7%) were associated with peri-implantitis. Conclusions: This study shows that the treatment of edentulous geriatric patients by immediate loading of implants with fixed rehabilitations is a clinically successful protocol but with a high prevalence of peri-implant diseases.

## 1. Introduction

Therapy with complete denture has become the conventional treatment choice for geriatric edentulous patients, supplying improved aesthetics and function. However, the progressive bone resorption of the alveolar ridge, because of total edentulism, can cause denture instability and retention, especially for the lower denture, reducing functional comfort and aesthetics. Patients wearing complete removable prostheses often complain about the inefficiency of their dentures, with an overall dissatisfaction with their prosthesis [1,2].

Dental treatment with immediate loading implant prostheses is recognized as a restorative treatment with high predictability and a favorable long-term prognosis in older people.

Several studies with overdentures and fixed complete prostheses have shown that implant treatment in older people can be a successful possibility for the rehabilitation of edentulous patients [3,4,5,6].

Complete-arch implant-supported fixed dental prostheses are widely accepted as a treatment option for edentulism, maintaining implant survival and success rates well above 90% [7]. Moreover, new implant and prosthetic designs and materials for the restoration of the edentulous arches are currently used [8,9]. In edentulous patients, the use of fixed implant-supported prostheses offers an effective solution for the restoration of the chewing function and aesthetics. This results in a notable enhancement in the patients’ quality of life, on both a personal and social scale [9,10].

These complete-arch fixed dental prostheses present favorable clinical outcomes with all loading protocols (conventional, early, and immediate) [11]. The transition from failing dentition to complete edentulism using the immediate installation of implant-supported fixed prostheses has always been a priority goal in geriatric prosthodontics. Immediate insertion of implants into fresh sockets with the placement of immediate provisional prostheses is reported in several studies with a high success rate [11,12,13]. Hence, reducing the healing period to loading would be of great benefit to edentulous patients [12,13,14,15,16].

Several research studies have provided evidence of the existence of both biological and technical complications in edentulous patients who undergo treatment with complete-arch fixed dental prostheses [17,18,19,20,21]. A retrospective study of 10 years reported a high prevalence (47.8%) of technical complications, such as screw loosening/fracture, ceramic chipping/fracture, and loss/fracture of acrylic teeth [18]. Several influencing factors for peri-implant mucositis and peri-implantitis are described in edentulous patients treated with totally fixed rehabilitations. For the development of peri-implant mucositis, the risk factors of plaque accumulation, residual cement excess, or smoking could be reported. For peri-implantitis risk factors such as a history of periodontitis, smoking, plaque accumulation, or noncompliance to recall or prosthetic design seem to have a great impact [19,20,21].

The objective of this clinical study was to evaluate the clinical results of immediate loading of implants for fixed rehabilitations in elderly edentulous patients.

## 2. Materials and Methods

### 2.1. Sample Description

This retrospective study was conducted on periodontal patients who had total or partial edentulism, necessitating the extraction of all remaining teeth, and on cases where immediate dental implant placement with immediate loading was part of the treatment plan. Ex-smokers were considered for the study if they had not smoked for at least 1 year, but if any patient had shorter periods, this was recorded. All the variables analyzed in the study were extracted from medical records; it is noteworthy that all the treatments were carried out by the same research teaching group.

Both surgery and prosthetic procedures were performed at the Faculty of Dentistry of the University of Seville, Spain, from March 2018 to June 2022. The study was carried out by the principles described in the Declaration of Helsinki [22] pertaining to research in humans.

The study was approved by the ethics committee of the University of Seville, and all the patients signed informed consent for treatment and participation in the study.

#### Inclusion and Exclusion Criteria

Among patients requiring dental treatment, the following inclusion criteria were established: adult patients with a well-maintained systemic health status (classified as ASA I or II) or effectively managed systemic conditions, who did not require bone regeneration procedures, and who were edentulous or required the extraction of remaining teeth. Based on the study by Palacios et al. [23], which analyzes bone loss and failure in a series of implants, we hoped to obtain at least 62 implants; however, we analyze all those who were placed with the same brand in this group of patients.

The exclusion criteria encompassed uncontrolled chronic systemic conditions (such as diabetes and cardiovascular disease), smoking at a rate exceeding ten cigarettes per day, coagulation disorders, alcohol or substance abuse, and the utilization of any medication or health condition that contraindicates implant treatment.

### 2.2. Diagnosis and Treatment Plan

**Figure 1:**
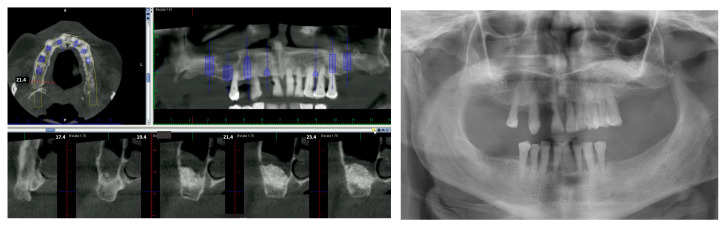
Panoramic radiograph and one of the cuts of planning using CBCT.

The patients were chosen after a detailed clinical history was performed and the patients agreed to participate in the study. Intermaxillary relationships, clinical images, and panoramic radiographs were assessed through diagnostic models (Figure 1). Whenever necessary, the patients were evaluated with CBCT (cone beam computer tomography) when required. Through planning, the number of implants was decided as well as their location based on bone availability and the most suitable prosthesis for the case.

### 2.3. Surgery Protocol

The patients were instructed to follow an antibiotic regimen, which included taking 500 mg of amoxicillin and 125 mg of clavulanic acid one hour before the procedure, followed by the same dosage every eight hours for seven days post-treatment. Additionally, a pain management plan involved taking 600 mg of ibuprofen every six hours for seven days. All the patients rinsed with chlorhexidine for two minutes before the intervention. They were encouraged to continue using the chlorhexidine mouthwash twice daily for a month. Local anaesthesia using articaine with adrenaline was administered to all the patients during the procedure. Patients who were smokers had to agree not to smoke 10 days post-surgery.

**Figure 2:**
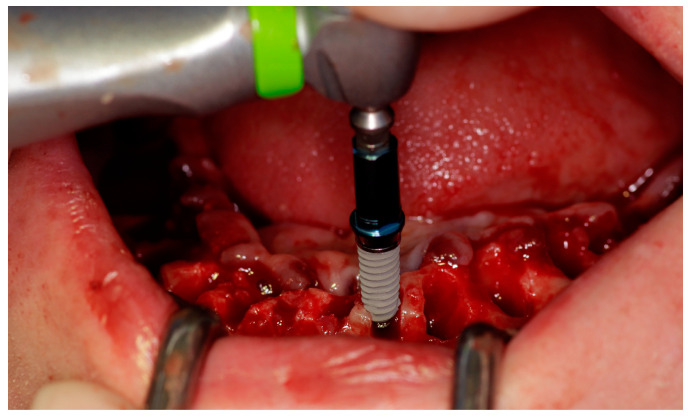
Clinical surgical protocol with implant placement.

Before surgery, all the patient’s remaining teeth (if any) were extracted, and after extraction, the patient rinsed again for 2 min with chlorhexidine 0.12%. A mucosal flap technique was employed, and the implants were placed at the chosen location according to a prosthodontically guided plan. The drilling protocol was the one recommended by the manufacturer (KYT dental implants^®^, Badajoz, Spain), and the minimum insertion torque was 30 Ncm. All the implants were inserted into fully healed bone and immediately after tooth extractions, using a one-stage surgical approach (Figure 2).

### 2.4. Prosthodontic Protocol

**Figure 3:**
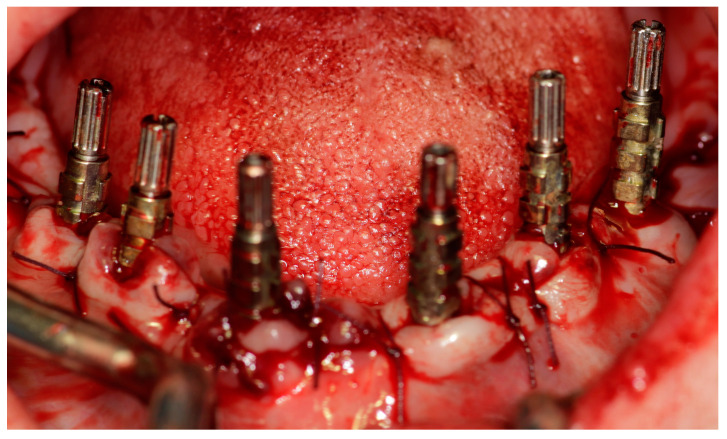
Clinical prosthodontic protocol for impressions.

After the placement of the implants, an immediate loading procedure was carried out. An impression method involving an open custom tray along with silicone material was performed (Figure 3). It was decided to place a hybrid prosthesis when replacement was deemed necessary, in addition to the lost teeth, a large amount of soft tissue, and/or lip support.

**Figure 4:**
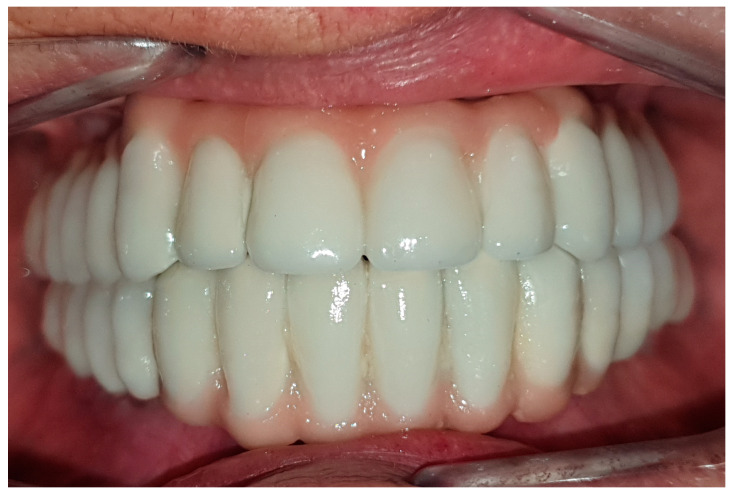
Placement of definitive implant-supported fixed prostheses.

After the impressions were made, prosthetic abutments were promptly positioned, and functional loading was carried out until an insertion torque of a minimum of 30 Ncm was reached. Immediately after the surgical procedure, provisional fixed prostheses supported by the implant were placed. After a control at 6 months, definitive metal-ceramic fixed rehabilitation was performed (Figure 4).

### 2.5. Follow-Up

A control visit was carried out at 7 and 21 days for postsurgical evaluation. Subsequently, check-ups were carried out at 3 and 6 months; in the latter, an orthopantomography was performed, and the definitive prosthesis was made. Subsequent to the placement of the definitive prosthesis, follow-up appointments were scheduled at 3-month and 6-month intervals, with annual visits thereafter. The success criteria were established as implant stability and the absence of radiolucency around the implant, mucosal suppuration, or pain. Marginal bone loss was determined by an intraoral digital radiograph taken perpendicularly to the long axis of the implant. The value was obtained by comparing the average measurement between the mesial and distal bone loss of the implant and comparing the result with the value recorded post-surgery.

### 2.6. Implant Characteristics

All the patients received KTX screw implants (KYT dental implants^®^, Badajoz, Spain). The implant surface underwent a treatment process involving sandblasting and acid etching to enhance its roughness, creating a microtopography. These implants were tissue-level and made of commercially pure (CP) titanium, grade IV, featuring an external hexagon connection.

### 2.7. Statistical Evaluation

Data analysis was conducted using SPSS 18.0 software (SPSS Inc., Chicago, IL, USA). Descriptive statistics were employed to present the results in the format of mean ± standard deviation. For statistical analysis, the chi-square test and two-way ANOVA with the U-Mann-Whitney test were utilized, and the significance level was set at *p* < 0.05.

## 3. Results

Two hundred ten implants were placed in 24 patients, 20 females (83.3%) and 4 males (16.7%), with an average of 8.7 implants/patient. Twenty-three patients were partially edentulous. Multiple extractions were performed, and the implants were inserted immediately post-extraction in the same operative session. In a completely edentulous patient, the implant insertion surgery was performed directly.

The average age of the patients was 67.9 ± 8.9 years old, ranging from 50 to 85. The mean age of the women was 67.4 ± 9.5 years, and the mean age of the men was 70.2 ± 4.2 years. The analysis of variance did not reveal any significant differences in this regard (ANOVA; *p* = 0.5772). Eleven patients (45.8%) were less than 65 years old, and 13 patients (54.2%) were more than 65 years old. The distribution of age categorized by sex shows that among the patients under 65 years of age there were 10 women (50%) and one man (25%), while among those over 65 years of age there were 3 men (75%) and 10 women (50%). These differences were not significant according to the chi-square test (*p* = 0.35964). All the patients had a previous history of periodontitis; 11 patients (45.8%) were smokers; and 11 patients (45.8%)—everyone in the over 65 age group—suffered chronic medical conditions (Table 1).

Among the cohort, there were eleven patients who smoked fewer than 10 cigarettes per day. A total of 94 implants (accounting for 44.7% of the total) were placed in this this group. The breakdown of patients in terms of age, gender, and medical history can be found in Table 2.

An average of 8.7 implants were placed in the patients (n = 210). The patients with a well-controlled medical history (hypertension, heart failure, diabetes) accounted for 11 (45.8%), and 97 implants (46.2%) were inserted in them. Neither in them nor in the rest of the variables analyzed using the Mann-Whitney U test were the results significant. (Table 3).

The 11 patients with medical pathology are distributed without statistical significance between men and women and between the two age groups (Table 4).

Of the 210 implants placed, 6 implants (2.8%) had a diameter of 3.3 mm, 45 (21.4%) had a diameter of 3.7 mm, 147 (70%) had a diameter of 4 mm, and 12 (5.7%) had a diameter of 4.5 mm. Twelve implants (5.7%) were 8.5 mm in length, 34 (16.2%) were 10 mm, 71 (33.8%) were 11.5 mm, 85 (40.7%) were 13 mm, and 8 (3.8%) were 15 mm. One hundred seventeen implants (55.7%) were inserted in the maxilla, and 93 implants (44.3%) were placed in the mandible. In four patients (16.7%), a sinus lift procedure in the maxillary sinus was carried out concurrently with the implant placement. Of the four patients who underwent direct sinus elevation, three were under 65 years of age (*p* = 0.19967), three were women (*p* = 0.62421), one was a smoker (*p* = 0.35964), and three had a medical history (*p* = 0.9967); there were no significant differences for any of the parameters.

Clinical follow-up was scheduled yearly with a mean period of 37.1 ± 14.6 months (ranging between 15 and 63 months). One implant was lost during the clinical follow-up, leaving a cumulative survival rate (CSR) for all the implants of 99.52%. One implant was lost in a female patient under 65 years of age, and this was in the anterior sector of the jaw.

Regarding the prostheses designed, a total of 33 fixed prostheses were placed in the 24 patients immediately after the teeth extractions. Eighteen fixed rehabilitations (54.5%) and 15 hybrid fixed prosthesis (45.5%) were inserted in the patients. In the maxilla, 15 fixed restorations (45.5%) and 3 total hybrid prostheses (9.1%) were performed. In the mandible, 12 total hybrid prostheses (36.4%) and 3 fixed restorations (9.1%) were performed. One patient (4.2%) showed some kind of technical complications (ceramic chipping) (Table 3).

The average marginal bone loss measured 1.33 ± 0.17 mm, with a range between 0.15 and 3.97 mm over the period from implant placement to the three-year follow-up assessment. These cases were not significant for the different variables analyzed.

Clinical follow-up presents some differences between the groups (Table 5). Some complications appeared in 11 patients during the follow-up period. One patient presented simultaneously with mucositis, peri-implantitis, and a ceramic fracture. Another patient presented peri-implant pathology and a lost implant. In Table 6, we present the most relevant data on these complications.

This series of patients reported a high prevalence of peri-implant diseases. During the follow-up period, 11 patients (45.8%) showed mucositis. Mucositis was significantly most frequent in patients with a higher clinical follow-up of 30 months (chi-square test, *p* = 0.01239); but it was not significant with any other parameter analyzed, including type of prosthetic rehabilitation (fixed or hybrid) with *p* = 0.604 and marginal bone loss (Table 7).

Twenty-five implants (11.9%) in 10 patients (41.7%) were associated with peri-implantitis (Table 8). Peri-implantitis occurred more often, with statistically significant variances observed among patients with chronic medical conditions (87.5%) according to the chi-square test (*p* = 0.0438). In smoking patients, peri-implantitis was also more prevalent (100%), although the chi-square test did not reveal statistically significant differences (*p* = 0.14766 and *p* = 0.58596, respectively).

However, as expected, peri-implantitis was more frequent in patients with more bone loss (*p* = 0.0116).

## 4. Discussion

The aim of this study was to examine the clinical results of edentulous individuals who underwent treatment with complete-arch fixed dental prostheses utilizing immediate impact loading. Immediate loading seems to be a viable choice in specific cases when carefully selected, aiming to prevent early implant-related failures. For geriatric patients, this approach may offer advantages such as shorter treatment periods, fewer surgical procedures, and a quicker transition from tooth extraction to prosthetic rehabilitation, potentially leading to increased patient satisfaction [24,25]. This retrospective clinical study evaluated implant and prosthetics success rates in patients treated with an immediate loading protocol with a follow-up of up to three years. The success rate of the implants and prosthodontics placed in this study yielded 99.5% and 96.9%; respectively.

Immediate loading is considered the treatment protocol in which a prosthetic reconstruction is attached to the implants within three days after the implant surgery. The main advantage of immediate loading is reduction in treatment time, which may explain the popularity of this functional loading. The effectiveness of this method in clinical practice relies significantly on various factors, including careful selection of patients, quality and quantity of available bone, number of implants and their initial stability, design of the prosthetic components, and occlusal loading. Implant initial stability is undoubtedly the most important of these factors. In implant dentistry, there is growing interest in shortening the time frame between implant placement and installation of a functional prosthesis, providing faster comfort as well as aesthetics to older patients [26,27].

The placement of implants immediately after tooth extraction has proven to be a predictable treatment strategy with a very high success rate [12,13,28,29]. However, alveolar ridge volume loss after tooth extraction is an irreversible process with horizontal and vertical dimensional changes [27,30]. The potential advantages of immediate restoration of implants placed in fresh extraction sockets have been widely reported, with similar clinical outcomes to implants placed in healed ridges [12,31,32,33]. In this study, any remaining periodontally compromised teeth were removed before implant placement. All the implants were inserted simultaneously, in fully healed bone and immediately in fresh sockets. No differences in survival or bone loss were found between them.

Several clinical studies have demonstrated favorable clinical outcomes of immediate implants in fresh sockets for full-arch restorations [32,33]. A retrospective study documented the long-term clinical efficacy of immediate restoration of both post-extraction and non-post-extraction implants supporting full-arch restorations [32]. The patients received a screw-retained provisional restoration within 48 h of surgery and a definitive screw-retained prosthesis within one year. Sixty-six patients received 494 implants distributed in 75 prostheses. The median follow-up was 86 months (range 82–168 months). Only three implants had failed at the last follow-up. All the implant failures occurred in patients who smoked. Implant survival was 99.6%. No difference in clinical success could be observed between post-extractive and non-post-extractive implants [32].

A recent study demonstrated that both immediate and delayed implant placements are sound options with predictable treatment outcomes for full-arch rehabilitations [34]. A total of 4519 records of implants were included. Immediate implants were significantly more frequently placed in the maxillary arch than in the mandible. The analysis of survival rates indicated that there were no notable distinctions between implant placements conducted immediately and those performed with a delay. The mean follow-up time was 32.27 months, during which 1.5% immediate and 1.1% delayed implants were removed. The placement of immediate implants achieved similarly high survival rates when compared to delayed implants placed in healed sites [34].

All the patients in this study showed a history of periodontitis. The immediate loaded fixed prostheses supported by implants can be a suitable treatment option for geriatric edentulous patients with a history of periodontitis, with high survival implant rates [35,36]. A retrospective clinical study reported the results of 119 patients treated with 146 prostheses over a total of 642 implants. The mean follow-up period was 41 months. The survival rates were found to be 98.3% at the implant level and 92.4% at the patient level. Mechanical issues were observed in 55 (37.7%) of the final prostheses, while biologic complications were identified in 318 (49.5%) implants. Significantly, smokers exhibited a lower survival rate compared to non-smokers. Bruxers had a significantly higher incidence of mechanical complications than non-bruxers [35]. In this work, we have not analyzed this aspect, but we have obtained a somewhat higher survival rate (99.5%) than in the study we are commenting on.

Other authors with standardized protocols for patients with severe periodontitis obtained similar results [36]. So, Slutzkey et al. [36] present a total of 84 axial and 46 tilted immediate implants that were inserted into the extraction sockets of 23 patients using a protocol involving four to six implants combined with ridge augmentation. Within 72 h, a temporary prosthesis was cemented to the implants, followed by the delivery of a permanent ceramic-metallic prosthesis after a six-month period. The 5-year survival rate of the straight and tilted implants was 100% and 97.8, and 5-year survival rate of the prosthetic implants was 100%.

This study showed the clinical outcomes of 33 fixed prostheses that were installed in 24 patients immediately following the extraction of their teeth. Eighteen fixed rehabilitations (54.5%) and 15 hybrid fixed prosthesis (45.5%) were installed in the patients. One patient (4.2%) showed some kind of technical complications (ceramic chipping). Several clinical studies reported favorable long-term evidence of fixed prosthetic rehabilitation by immediate loading of implants. This clinical protocol can be a general treatment strategy for fully edentulous arches in geriatric patients [37,38,39]. However, the available evidence suggests that a higher incidence of biological and technical complications is reported with complete-arch fixed dental prostheses [33,40,41].

In a clinical study, the prevalence of both biological and mechanical complications was assessed in edentulous patients who received complete-arch implant-supported fixed dental prostheses [41]. The study included a total of 44 prostheses, supported by 268 implants, in 30 patients, with an average follow-up duration of 4.8 years. Among these prostheses, 18 were made of zirconia-ceramic, while 26 were titanium-ceramic. The cumulative success rate was 99.3% for the implants and 92.5% for the prostheses. The most commonly observed biological complication was peri-implant mucositis, occurring in 4.5% of the cases, followed by peri-implantitis (3.0%). The most common mechanical complication was ceramic chipping (45.5%), followed by crown debonding (13.6%) and framework fracture (4.5%). The presence of a cantilever and a maxillary arch was significantly associated with mechanical complications [41].

This study reported a high prevalence of peri-implant diseases in the patients (50%). During the follow-up period, 11 patients (45.8%) showed peri-implant mucositis, and 10 patients (41.7%) were associated with peri-implantitis. Several clinical studies have demonstrated an important prevalence of peri-implant diseases in edentulous patients treated with totally fixed restorations over immediate implants [17,18,19]. A review reported the analysis of a total of 18 studies that were included. The prevalence of peri-implant mucositis in fully edentulous patients was 57%, corresponding to 47% at the implant level. In fully edentulous patients, the prevalence of peri-implantitis was found to range between 1.5% and 29.7% of patients and between 2.1% and 20.3% of the implants [42].

In the present study, peri-implant mucositis was significantly most frequent in patients with a higher clinical follow-up of 30 months. Moreover, mucositis was most prevalent in patients treated with upper ceramic fixed rehabilitation (53.3%) and lower hybrid prosthesis (50%). The incidence of mucositis, in edentulous patients rehabilitated with implant-supported prostheses, is strongly related to the long-term plaque accumulation around implants supporting metal-acrylic resin prostheses and metal-ceramic prostheses [41,43,44]. Peri-implant mucositis occurred more often under maxillary full fixed prostheses, while peri-implantitis appeared more common around mandibular implants [44].

Peri-implant mucositis is assumed to precede peri-implantitis. Hence, scientific evidence indicates that patients diagnosed with peri-implant mucositis may develop peri-implantitis, especially in the absence of maintenance care. The progressive loss in the supporting bone characterizes the progression from peri-implant mucositis to peri-implantitis, clinically identified by bleeding and/or suppuration and an increased pocket depth. Peri-implantitis develops early, and bone loss can progress with an individual pattern related to susceptibility and risk factors (i.e., history of periodontitis, smoking) [45,46].

Some studies have reported that peri-implant diseases may develop over the long-term follow-up of edentulous patients treated with totally fixed rehabilitations by immediate loading of dental implants [12,19,20,21,32,42]. In the present study, 25 implants (11.9%) in 10 patients (41.7%) were associated with peri-implantitis. These findings are corroborated by a retrospective study that assessed the long-term peri-implant status of patients who underwent treatment with immediately loaded full-arch prostheses [21]. In this study, a total of 378 implants were placed in 56 patients, with 40 upper and 32 lower arches receiving restoration. Additionally, 16 patients received bimaxillary rehabilitation. The average follow-up period was 50 months, and the prevalence of peri-implantitis among implants and patients was 14.3% and 50%, respectively. Mucositis was observed in 56.9% of the implants and affected 50% of the patients. The rate of success was 95.5% for implants and 80.4% for patients [21].

Several risk factors, such as the history of periodontitis, smoking, systemic diseases, number of implants, and prosthodontic design, are related to the incidence of peri-implantitis in patients treated with immediately loaded implants with totally fixed rehabilitation [12,21,35,40,44,47]. However, in the present study, no statistical significance was found on peri-implantitis about demographic and clinical findings of geriatric patients (Table 8). Peri-implantitis was more frequent in non-smoker patients and patients without medical conditions.

## 5. Conclusions

The clinical results of this retrospective study, although they must be evaluated with caution as it is not a prospective study, reported that the treatment of edentulous geriatric patients by immediate loading of several implants with fixed rehabilitations is a clinically successful protocol. The favorable evolution of this technique is highly dependent on factors such as patient selection, a correct diagnosis of bone quality and quantity, the insertion of enough implants with primary stability, and an adequate prosthetic design. However, some biological complications, mucositis and peri-implantitis, are present in an important rate of patients but do not affect implant survival.

## Figures and Tables

**Table 1:** Demographic and clinical variables of patients (n = 24).

Variables		
Age	≤65 years	>65 years
	11 (45.8%)	13 (54.2%)
Sex	Men	Women
	4 (48.1%)	20 (51.9%)
Tobacco	Smokers	Nonsmokers
	11 (45.8%)	13 (54.2%)
Systemic diseases	YES	NO
	11 (45.8%)	13 (54.2%)
Clinical follow-up	<30 months	>30 months
	11 (45.8%)	13 (54.2%)

**Table 2:** Smoker patients (n = 4, 14.7%).

Variables			*p* Value
Age	≤65 years	>65 years	0.97267
	5 (33.3%)	6 (46.2%)	
Sex	Men	Women	0.35964
	1 (25%)	10 (50%)	
Systemic diseases	YES	NO	0.09322
	3 (27.3%)	8 (61.5%)	
Clinical follow-up	<30 months	>30 months	0.39174
	4 (36.4%)	7 (63.6%)	

Note: *p*-values below 0.05.

**Table 3:** Distribution of the number of implants (n = 210).

Variables			*p* Value
Age	≤65 years	>65 years	0.3273
	104 (49.2%)	106 (50.8%)	
Sex	Men	Women	0.4508
	39 (81.4%)	171 (18.6%)	
Tobacco	Smokers	Nonsmokers	0.5931
	94 (44.7%)	116 (55.3%)	
Systemic diseases	YES	NO	0.5727
	97 (46.2%)	113 (53.8%)	
Clinical follow-up	<30 months	>30 months	0.3273
	87 (41.4%)	123 (58.6%)	
Lower prosthesis	Hybrid	Totally	0.7143
	69 (25.8%)	24 (74.2%)	
Upper prosthesis	Hybrid	Totally	0.7163
	18 (14.4%)	99 (84.6%)	

Note: *p*-values below 0.05. Hybrid: hybrid fixed prosthesis; Totally: totally fixed rehabilitations.

**Table 4:** Patients with medical pathology (n = 11, 45.8%).

Variables			*p* Value
Age	≤65 years	>65 years	0.4307
	6 (54.22%)	5 (38.5%)	
Sex	Men	Women	0.8546
	2 (18,184%)	9 (81.8%)	

Note: *p*-values below 0.05.

**Table 5:** Distribution of patients according to follow-up.

Variables				*p*-Value
Follow-up		15–30 Months	30–63 Months	0.6902
		11 (45.8%)	13 (54.2%)	
Age		≤65 years	>65 years	
	<30 Months	2 (18.3%)	9 (69.2%)	0.0123
	≥30 Months	9 (69.2%)	4 (30.8%)	
Sex		Men	Women	
	<30 Months	3 (27.2%)	8 (72.7%)	0.1996
	≥30 Months	1 (7.6%)	12 (92.3%)	

Note: *p*-values below 0.05.

**Table 6:** Distribution of patients with complications during follow-up (n = 11, 45.8%).

Variables			*p* Value
Age	≤65 years	>65 years	0.7291
	5 (45.5%)	6 (54.5%)	
Sex	Men	Women	0.4589
	2 (18.1%)	9 (81.8%)	
Smoking	YES	NO	0.18827
	3 (27.3%)	8 (61.5%)	
Systemic diseases	YES	NO	0.04462 *
	7 (63.6%)	4 (36.3%)	

Note: *p*-values below 0.05. *: Statistically significant.

**Table 7:** Distribution of patients (n = 11) with mucositis.

Variables				
Age		≤65 years	>65 years	*p*-Value
	Patients	6 (54.5%)	5 (38.5%)	0.4307
			
Sex		Men	Women	
	Patients	1 (25%)	10 (50%)	0.3596
			
Smoking		Smokers	Nonsmokers	
	Patients	4 (36.4%)	7 (53.8%)	0.3917
			
Systemic diseases		YES	NO	
	Patients	6 (18.2%)	5 (69.2%)	0.4307
			
Clinical follow-up		<30 months	>30 months	
	Patients	2 (18.2%)	9 (69.2%)	0.0123 *
			
Marginal bone loss		Mucositis	No mucositis	
		1.64 ± 0.98 mm	1.07 ± 0.41 mm	0.0874
Lower prosthesis		Hybrid	Totally	0.0896
		6 (50%)	1 (33.3%)	
Upper prosthesis		Hybrid	Totally	0.6047
		0 (0%)	8 (53.3%)	

Note: *p*-values below 0.05. *: statistically significant. Hybrid: hybrid fixed prosthesis; Totally: totally fixed rehabilitations.

**Table 8:** Distribution of patients (n = 10, 41.6%) and implants (n = 25, 11.9%) with peri-implantitis.

Variables				
Age		≤65 years	>65 years	*p*-Value
	Patients	3 (27.2%)	7 (53.8%)	0.1925
	Implants	13 (12.5%)	12(11.3%)	0.7472
Sex		Men	Women	
	Patients	2 (50%)	8 (40%)	0.2610
	Implants	10 (8.7%)	15 (25.6%)	0.0355 *
Smoking		Smokers	Nonsmokers	
	Patients	3 (27.2%)	7 (53.8%)	0.1925
	Implants	8 (8.5%)	17 (14.6%)	0.3710
Systemic diseases		YES	NO	
	Patients	4 (36.3%)	6 (46.1%)	0.7685
	Implants	13 (13.4%)	12 (10.6%)	0.6922
Clinical follow-up		<30 months	>30 months	
	Patients	4 (36.3%)	6 (46.1%)	0.7685
	Implants	14 (16.1%)	11 (8.9%)	0.5127
Marginal bone loss		Peri-implantitis	No Peri-implantitis	
		2.27 ± 1.07 mm	1.08 ± 0.44 mm	0.0116 *
Lower prosthesis		Hybrid	Totally	0.7703
		6 (50%)	1 (33.3%)	
Upper prosthesis		Hybrid	Totally	0.3961
		1 (33.3%)	5 (33.3%)	

Note: *p*-values below 0.05. *: Statistically significant.

## Data Availability

Data sharing not applicable.
